# Qualitative and Quantitative Investigations on the Failure Effect of Critical Fissures in Rock Specimens under Plane Strain Compression

**DOI:** 10.3390/ma16020611

**Published:** 2023-01-08

**Authors:** Jiaqi Wen, Lei Tang, Shenghang Zhang, Qibing Zhan, Yukun Wang

**Affiliations:** 1 State Key Laboratory of Hydrology-Water Resources and Hydraulic Engineering, Nanjing 210029, China; 2 Department of Materials and Structures, Nanjing Hydraulic Research Institute, Nanjing 210029, China; 3 School of Water Resources and Hydropower, Wuhan University, Wuhan 430072, China; 4 School of Civil Engineering, Tianjin University, Tianjin 300350, China

**Keywords:** fissure of rock, failure analysis, plane strain compression (PSC), rock-bearing capacity index (RockBCI), discrete element method (DEM)

## Abstract

To investigate the failure effects of critical fissures in rock specimens subjected to plane strain compression (PSC), five types of internal fissures in rock specimens were designed and twelve PSC tests were conducted for two lithologies based on the discrete element method (DEM). The results were analyzed in terms of the fracture mode, data characteristics, and crack evolution. The results indicated the following. (1) The rock samples with a critical fissure under PSC showed a weak face shear fracture mode, which was influenced by lithology, fissure angle, and fissure surface direction. (2) There were four critical expansion points (CEPs) of axial stress of the rocks under PSC, which were the stage signs of rock materials from local damage to complete fracture. The rock-bearing capacity index (RockBCI) was further proposed. (3) The bearing capacity of rock samples with horizontal fissures, fissures whose angles coincided with the fracture surface, and fissures whose surface was perpendicular to the lateral confine direction was the worst; their BCI^2^ values were found to be 80.6%, 70.8%, and 56.9% of the rock samples without any fissures, respectively. The delayed fracture situation under PSC was identified and analyzed. (4) The crack evolution followed the unified law of localization, and the fissures in the rocks changed the mode of crack development and the path of the deepening and connecting of crack clusters, as well as affecting the time process from damage to collapse. This research innovatively investigated the behavior characteristics of rock samples with a fissure under PSC, and it qualitatively and quantitatively analyzed the bearing capacity of rock mass from local damage to fracture.

## 1. Introduction

The mechanical analysis of surrounding rock in underground engineering, such as hydraulics, mining, and traffic tunnels, can typically be viewed as a plane strain problem. For the underground engineering of what can be regarded as an infinite-length tunnel, the deformation of surrounding rock in the axial direction can be negligible and the transverse deformation perpendicular to the axial direction is the primary concern. With the traditional compression test of rock materials from the pre-peak, peak, and post-peak stages of a stress–strain curve, the mechanical responses of rock materials and underground engineering surrounding rock mass can be increasingly differentiated. The parameters and constitutive relations obtained from regular tests of rock materials greatly deviate when applied to engineering practice [1,2]. Therefore, the compression conditions of rock mechanics tests must be updated based on actual situations of underground engineering.

**Figure 1:**
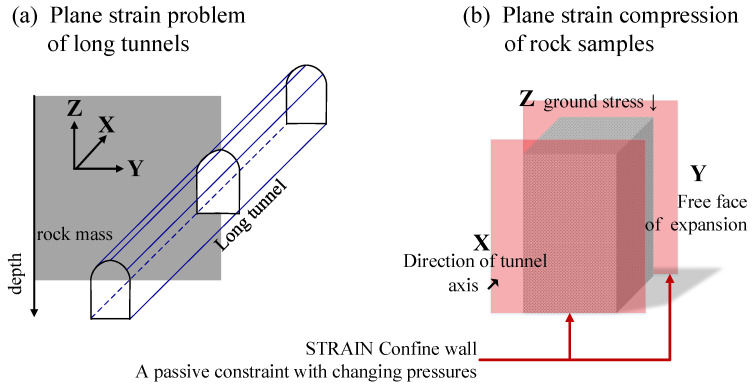
Plane strain problem of tunnels and PSC test of rock samples.

The plane strain compression (PSC) test of rock samples can be used to describe the stress state of the tunnel surrounding a rock [2], as shown in Figure 1, where the Z-loading direction corresponds to the vertical ground stress, the X-strain confine direction corresponds to the tunnel axis direction, and the Y-free direction corresponds to the transverse hollow surface of the tunnel. Investigating the ‘bearing–deformation–damage’ characteristics of rock materials subjected to PSC is of great theoretical and practical importance.

The PSC conditions are different from those of conventional biaxial compression (CBC). The constraint condition of CBC is ‘Stress Servo’, an active constraint with a stable pressure. The constraint condition of PSC is ‘Strain Confine’. During the axial loading of PSC, a rock sample generates lateral strain that compresses the confined device, so the stress on it is a passive constraint with varying pressures. The changing stress on a laterally confined device is associated with the lithology and fracture process of rock samples. Currently, PSC tests are most common in geotechnical contexts [3,4,5] and other investigations for metallic material properties [6,7,8], and there have been few PSC test studies for rock materials. Relevant studies involve the validation of rock PSC test benches, the observation of rock strain localization in simulation tests [9,10], and the analysis of the fracture mechanics of rock-like materials [11,12].

The reason that there are few PSC tests for rock materials is that it is difficult to ensure the strict lateral strain confine condition of the rock PSC physical test, which requires the confining device to have a strong capacity to restrict the deformation of a rock sample in a particular direction under vertical loading. According to a Chinese patent search, Yin et al. [13] invented a PSC apparatus for rock materials, and they noted that although the PSC apparatus could be used to obtain some parameters, it had many limitations, such as the complicated installation of deformation sensors, poor accuracy, and an inability to derive post-peak data. Additionally, Tang [14] developed a PSC test bench for rock materials that was cast from high-strength concrete reinforced with built-in channel steel; it still had limitations, including a narrow monitoring space, the inability to modify the distance between the sides of the bilateral confining device, and the need for other media to fill the gap between the lateral confining device and the rock samples. The computational method can be used overcome the equipment limitations of PSC physical tests, allowing for the rapid achievement of optimal PSC conditions and the collection of comprehensive monitoring information. This study used the discrete element method (DEM) to conduct PSC tests. The DEM is significantly superior to the traditional finite element method (FEM) for investigating rock crack development [15,16,17].

An actual rock mass consists of rock materials and many fissures. Widespread fissures in a rock mass can significantly affect mechanical properties such as strength, deformation, and fracture mode. Building a reliable fracture network model is the basis of the stability studies of tunnels. However, it is impossible to develop an actual rock mass model that includes all fissures, and the presence of numerous fissures makes it extremely difficult to analyze the mechanical properties of a rock mass. Therefore, it is urgent to identify the key fissure that significantly impacts the mechanical properties of a rock mass [18]. First, researchers examined the failure effect of different types of rock material fissures, such as horizontal, vertical, cross, and combined fissures [19,20]; fissures of various lengths [21]; fissures with varying opening degrees [22]; and fissures with different dip angles [23]. Researchers also investigated the failure impacts of rock samples with fissures exposed to different compression conditions, such as uniaxial compression [19,20,21,22,23], dynamic loading [18], triaxial compression [24], and the condition of first axial loading and then relieving confining pressure [25]. PFC of the DEM was applied in almost all of the abovementioned studies. In contrast, few studies have reported on PSC with rock materials, and the damage and fracture behavior of rock samples with fissures under PSC has not been effectively investigated and analyzed. The research results of this study will compensate for such research gaps. The contents of this research are as follows:

a. A series of rock samples with a particular fissure were individually designed, and 12 PSC tests were conducted for two kinds of lithology based on the DEM.
b. The fracture mode, crack development, and evolution laws of various rock samples with particular fissures were qualitatively evaluated.
c. Quantitative analysis was performed to explore the characteristics of the monitoring data of various specimens with certain fissures, and the following relevant quantitative indicators were proposed: the rock-bearing capacity index (RockBCI) and the time ratio index (TRI).

## 2. Materials and Methods

### 2.1. Methods

The particle discrete element method (PDEM) is widely used in geotechnical engineering. It has great superiority in building geotechnical material and structure models, establishing test conditions, and monitoring the damage and fracture of rock materials. In contrast to the continuous medium mechanics calculation method, the PDEM discretizes a material into many particles with varying diameters, a high stiffness, and mutual contact, and it simulates the interaction between the particle medium and its motion law. The PDEM can be convenient for dealing with discontinuous medium mechanical problems, reflecting the different physical relationships of a multi-phase medium. It can effectively simulate the cracking, separation, and other discontinuous phenomena of a medium, thus reflecting the mechanism, process, and results of mechanical phenomena. The traditional FEM cannot represent the complex interaction between particles and their highly nonlinear behavior, nor can it depict the flow deformation characteristics of bulk materials. The fast Lagrangian method (FLM) is also common in geotechnical engineering, and it is insufficient for evaluating overall stability due to its reliance on yield criterion, which can only determine the yield damage of local units.

**Figure 2:**
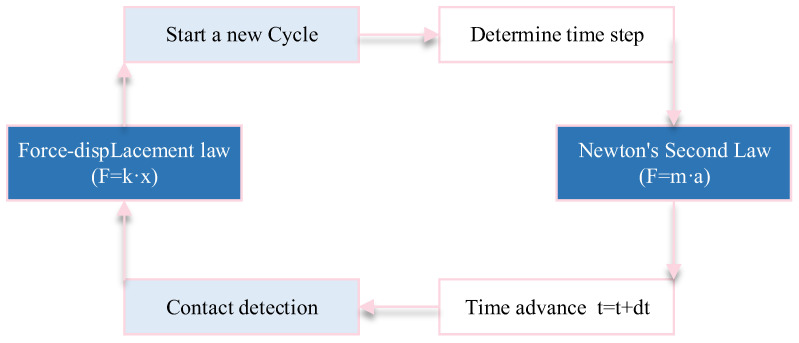
The basic cycle process of PFC.

PFC is a credible technical tool that is widely used in geotechnical studies [26,27,28,29]. The basic computational principle of PFC is based on the ‘force–displacement law’ and ‘Newton’s second law’, which is mainly solved in an iterative method using an explicit finite difference technique [30], as shown in Figure 2. The two laws are applied to particles and contacts between particles, respectively. ‘Newton’s second law’ is utilized to determine the state of motion of each particle caused by the contact and physical forces acting on the particles, while the ‘force–displacement law’ is employed to update the contact forces induced by the relative motion at each contact. The overall physical performance of a particle assemblage heavily depends on the contact properties, and the parallel bonded contact model (PBCM) can more accurately simulate a rock’s mechanical properties [31,32].

### 2.2. Rock Model

Two different lithologies, granite and sandstone, were utilized in this study. The granite samples were from Jinzhou City, Liaoning Province of China, and belonged to Mesozoic Yanshanian granite intrusion, which is grey-white, medium-coarse-grained biotite granite. The sandstone samples were from the Xikeng Tunnel of the Zhejiang Liquefied Natural Gas Pipeline Network Project of China. These two lithological samples are common geological materials in underground engineering.

The parameters of the PFC model are inconsistent with those of actual rock materials. It is suggested that the response calculated by the PFC model should be made consistent with the results of the standard uniaxial compression laboratory test of rock samples by continuously modifying the model parameters. There are parameter calibration rules that have been proven to be correct in practice and can be effectively used for PFC model parameter calibration. CUI [33] established empirical expressions between a single model parameter and a real parameter through fitting analysis and clarified the effect of different model parameters on rock mechanical performance response. The main conclusions were as follows: The ‘effective modulus’ mainly affects the elastic modulus of rock; the ‘stiffness ratio’ has a significant influence on the compressive strength, elastic modulus, and Poisson ratio of rock; and ‘the tensile strength, the cohesion and the friction angle of PBCM’ mainly affect the compressive strength of rock. Table 1 summarizes the specific calibration rules.

**Figure 3:**
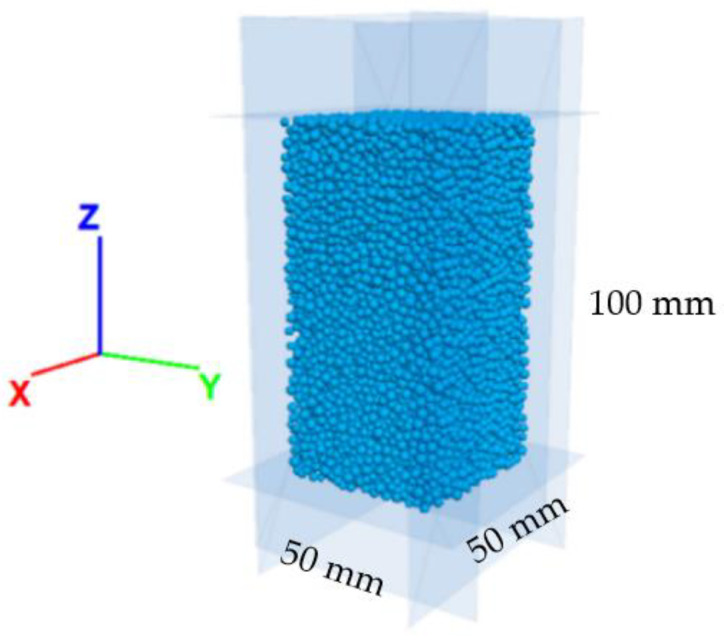
Rock model by Particle Flow Code.

Two 50 × 50 × 100 mm cuboid models based on PFC were established and given granite and sandstone parameters, as shown in Figure 3. The laboratory standard uniaxial compression test results of these two lithologies were obtained from references [33,34]. The model parameters of the two lithologies were derived according to the rules in Table 1 using laboratory findings as the target parameter. Table 2 shows the parameter calibration test results. These demonstrate that the mechanical response of the established PFC model was consistent with the laboratory test results, and the maximum error was less than 6%.

### 2.3. Test Design

The smooth joint model (SJM) or ball deletion method (BDM) is usually applied to represent fissure characteristics in the numerical simulation of a rock mass with fissures. Because the BDM leads to the non-conservation of the model mass, this paper used the SJM to set the property of the fissures.

Based on PFC, an inner fissure in the 3D rock model presented as a “circular disk” with a certain thickness. A definite fissure with a diameter of 20 mm was placed at the center of the rock samples. The fissure was assigned with the SJM, in which the normal stiffness was 3.0 GPa, the tangential stiffness was 3.0 GPa, the friction coefficient was 0.7, and the tension strength, cohesion, and friction angle were all 0. A total of 12 test conditions were designed: two tests of rock samples without any fissures and ten tests of rock samples with fissures. There were five types of fissures in the granite and sandstone rock samples under PSC:

Type 1—the fissure surface was 90° to the horizontal and was parallel to the strain confine wall.

Type 2—the fissure surface was 45° to the horizontal and was parallel to the strain confine wall.

Type 3—the fissure surface was 90° to the horizontal and was perpendicular to the strain confine wall.

Type 4—the fissure surface was 45° to the horizontal and was perpendicular to the strain confine wall.

Type 5—the fissure surface was 0° to the horizontal.

**Figure 4:**
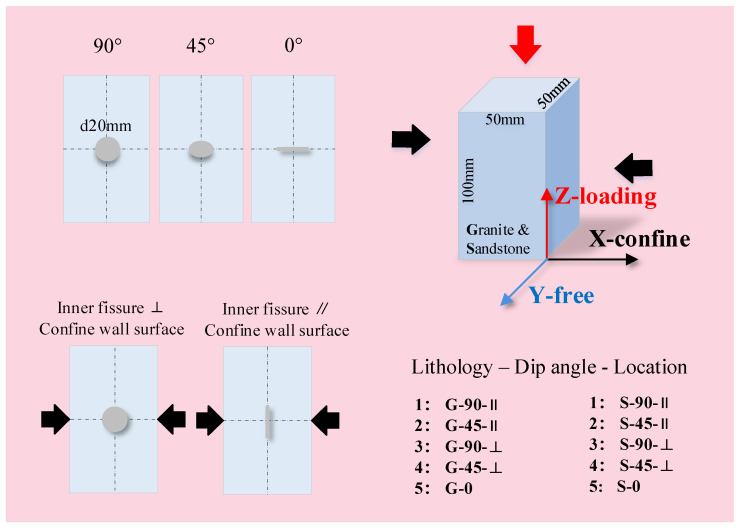
Test design details.

Figure 4 details the five types of fissures: type 1 and type 3 fissures were vertical, type 2 and type 4 fissures were declining, and type 5 fissures were horizontal. Type 1 and type 2 fissures were parallel to the confine wall, and type 3 and type 4 fissures were perpendicular to the confine wall. Z denotes the compression loading direction, X denotes the confine direction, and Y denotes the free direction.

A total of 12 tests were conducted. During the tests, the axial stress and axial strain of the samples were monitored in real time, especially the strain of the free direction and the development trend of generated cracks. The following contents were highlighted:

(a) Fracture Modes: The differences and causes of the fracture mode and crack development in two kinds of rock samples with various types of fissures under PSC.
(b) Date Characteristics: The relationship between the bearing capacity, expansion of the free direction, and crack number of rock samples under PSC, as well as the impact of the different types of fissures on the bearing capacity after the peak stress of the rock.
(c) Crack Evolution: The effect of various types of fissures on the process of the rock specimens from local damage to complete fracture under PSC.

## 3. Results

### 3.1. Fracture Modes

**Figure 5:**
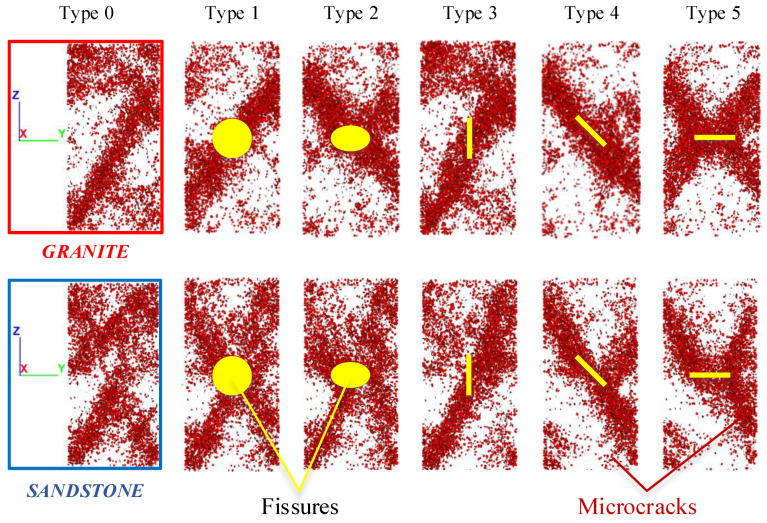
Fracture modes of rock samples under PSC.

Figure 5 shows the X-direction view results of all rock samples after completing the tests. The fracture of the granite and sandstone rock samples with the type 0 fissure presented a shear failure mode along the Z and Y directions. The fracture angle of the granite sample was about 30° toward the axial direction, and its shear surface covered more than 2/3 of the sample. The fracture angle of the sandstone sample was about 45°, with its shear surface covering about 1/2 of the sample. Outside the fracture surface, cracks developed extensively at the samples’ upper and lower ends.

Regarding the rock samples containing a fissure type, this study individually analyzed three factors.

Lithology: The two lithological rock samples with fissures were broken along the Z and Y directions. The typical differences in the fracture mode were that the granite samples had a shorter fracture face and a larger damage angle. Even though the sandstone samples had a longer fracture face and a smaller damage angle, the fracture face tended to penetrate the specimen in the vertical direction. For example, in the granite sample with a type 4 fissure, the fracture shear plane coincided with its 45° fissure, whereas in the sandstone sample with the same fissure, the fracture plane was along the line connecting the tip of the 45° fissure to the specimen’s end.

Fissure dip angle: Only the fracture surface of the rock sample with a type 4 fissure coincided with its fissure surface, while the fracture surface of other rock samples did not follow their fissure surface. The damage criteria of rock structural surface can be expressed as follows:

(1) $$\sigma_{1}-\sigma_{3}=\frac{{2c}_{j}+2\sigma_{3}\tan\varphi_{i}}{\left(1-\tan\varphi_{j}\cot\beta \right)\sin2\beta }$$

where $c_{j},\;\;\varphi_{j}$ are constant parameters of the rock materials and β is the fissure dip angle.

For fissure types 3 through 5, $\sigma_{3}$ was 0. Hence, when $\varphi_{j}<\beta <90{}^{\circ}$ was satisfied, the rock sample was fractured along the fissure surface; otherwise, the fracture was decided by the rock material itself.

(a) It was found that 90° fissures had the smallest effect on the original fracture mode. For instance, the granite sample with a type 3 fissure had almost the same fracture mode as the type 0 sample. Moreover, the shear fracture angle of the granite with a type 1 fissure had the same fracture mode as the type 0 sample, but the fracture surface did not occur along the fissure face.
(b) It was found that 45° fissures changed the original fracture angle and directly caused the weak face to fracture under shear. The fracture surface of the granite samples was along the 45° fissure, while the fracture surface of the sandstone samples did not form entirely along the 45° fissures but at an angle below 45°.
(c) It was found that 0° fissures were also the dominant factor of weak surface shear damage. Cracks were developed from the 0° disk end to the sample end to generate the shear fracture surface.

**Stress state:** The fissure surface’s direction significantly impacted the fracture mode of the rock samples under PSC, which caused a variation in the fissures’ stress states. Similar to the granite samples with fissure types 1 and 3, the fracture mode was almost unchanged when the fissure surface was perpendicular to the confine wall. When the fissure surface was parallel to the confine wall, different fracture modes were formed.

**Figure 6:**
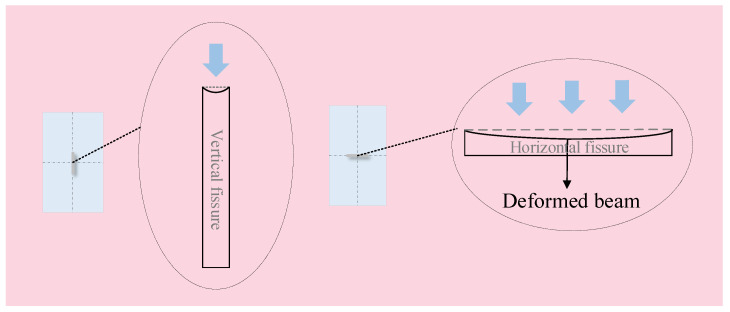
Simplified force analysis of a fissure in a rock sample.

A fissure with a certain thickness in a rock sample could be simplified as a solid-supported beam model, as shown in Figure 6. The upper part of the horizontal fissure could be regarded as a beam under uniform load under the assumption that the rock sample was only subjected to axial loading. Hence, when the beam was deformed, it led to apparent tensile stress in the middle and compressive stress on both sides. Compared with the horizontal fissure, the beam effect formed by the vertical fissure was significantly reduced and there was no apparent stress around it. Therefore, when the PSC’s two-direction stress (from Z and X) acted vertically on the fissure surface, the fissure significantly affected the fracture mode of the rock samples. On the contrary, the influence was small when the PSC’s two-direction stress was parallel to the fissure surface. Furthermore, the vertical stress (Z) was much greater than the confine wall stress (X). Thus, it can be judged that the influence of the stress state on the five fissure types was ranked from high to low as 5 > 2 > 4 > 1> 3.

In summary, the lithology, fissure angle, and stress state affected the fracture mode of the PSC specimens. The lithology and fissure angle affected the fracture angle and the potential of fracturing along the fissure surface. The fissure surface’s direction determined the fissure’s stress state, so it also determined whether there was obvious stress concentration around the fissure that affected the rock samples’ fracture process and bearing capacity.

### 3.2. Data Characteristics

**Figure 7:**
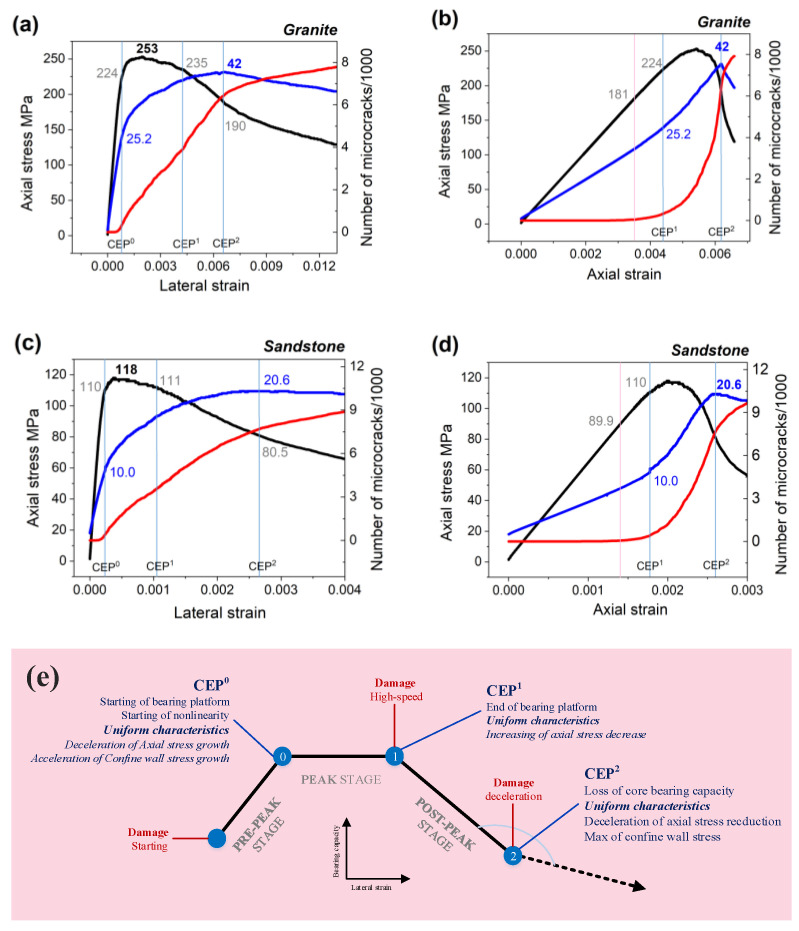
Data curve and its characteristic points.

Figure 7 shows the test results of two lithologic samples without any fissures, which are from the ‘type 0’ fissure in Figure 4. The ordinate in Figure 7 contains three kinds of data: the stress of the loading wall, the stress of the strain confine wall, and the number of microcracks. The abscissa in Figure 7a,c is the lateral strain from the Y-free direction, and that in Figure 7b,d is the axial strain from the Z-loading direction. The test process was realized via the axial loading of upper and lower walls at a constant rate. Therefore, the axial strain uniformly varied with time. However, the lateral strain did not uniformly change with time, which was small before the peak and significantly increased at the peak and post-peak stages. There were two different data curves: Figure 7a,c, based on the lateral strain, more clearly illustrates the post-peak bearing and damage characteristics of the rock samples, and Figure 7b,d (according to the axial strain) demonstrates the pre-peak stress change and initial damage characteristics. Figure 7 reveals the following.

**Stress of axial wall:** At the peak stress stage, the axial stress had a bearing platform process, in that with the increase in the lateral strain, the axial stress did not significantly decrease over time. At the post-peak stage, the axial stress rapidly and then slowly decreased, reflecting the ‘strain softening’ of the rock materials.

**Stress of the confine wall:** The confine wall stress reached its maximum at the post-peak stage of axial stress, and it demonstrated a three-stage process of “linear growth→accelerated growth→reaching the peak then decreasing” with axial strain. The confine wall stress was the passive pressure caused by the lateral expansion of the rock samples due to the axial loading. Its variation depended on the material properties of the rock sample and the damage and fracture of the rock mass during the test.

**Microcrack development:** The microcrack growth curve had an initiation position at the linear stage before the peak of axial stress. The maximum value of the microcrack growth rate was reached at the peak and post-peak stages. Then, the microcrack development showed a deceleration trend, and there was an obvious deceleration turning point after the peak, indicating the collapse of the core-bearing capacity of the sample.

Accordingly, there were three critical points in the axial stress curve: (1) deceleration point of stress growth (starting point of the bearing platform), (2) increase point of stress reduction (endpoint of the bearing platform), and (3) deceleration point of stress reduction.

There were two critical points in the confine wall stress curve: (1) acceleration point of stress growth and (2) the max value point.

There were three key points in the microcrack development trend: (1) start point of crack development, (2) the fastest crack development point, and (3) the deceleration point of crack development.

These key points overlapped, forming four critical key points with unified characteristics, as shown in Figure 7e.

1. **Damage starting point (DSP):** At this point, the axial and confine wall stress were still in a linear growth stage.
2. **Initial critical expansion point (CEP^0^):** At this point, the axial stress entered the deceleration growth stage with the lateral strain, which was the starting point of the bearing platform at the peak stage, indicating the beginning of the nonlinear deformation stage of the rock samples. At the same time, the confined wall’s stress began to rapidly increase, and the microcrack development also started to significantly rise.
3. **First critical expansion point (CEP^1^):** At this point, the axial stress began to quickly drop, which was the end of the bearing platform. Simultaneously, the microcrack growth rate reached the maximum.
4. **Second critical expansion point (CEP^2^):** At this point, the decrease in axial stress at the post-peak stage significantly slowed down. At the same time, the stress of the confine wall reached its maximum value, and the microcrack development began to significantly decelerate.

These critical expansion points (CEPs) have important physical significance. They represent the periodic mechanical performance of the rock specimens under PSC from bearing to damage and collapse. The confine wall’s stress variation can reflect the rock samples’ damage–bearing–collapse process. The rock sample exerted its bearing capacity when the confine wall stress increased from the acceleration point to the maximum point. When the confine wall stress reached the maximum value, the core-bearing capacity of the rock sample collapsed.

It is known that CEP^1^ and CEP^2^ are the periodic mechanical signs of a rock sample from damage to collapse. Therefore, the later the CEPs appear, the later the bearing capacity of a sample decreases or the collapse of the bearing capacity occurs. When the lateral strain at the CEPs is significant and a sample’s axial stress is small, the bearing capacity of the rock sample improves. On the contrary, when the lateral strain at the CEPs is small and a sample’s axial stress is large, the bearing capacity of the rock sample is reduced. Thus, the bearing capacity of a rock sample is in direct proportion to the CEPs’ abscissa and in inverse proportion to the CEPs’ ordinate.

Supposing $\sigma_{\max}$ is the axial peak stress, α,  β are the bearing capacity coefficients of the CEPs, and $e_{1}$ and $e_{2}$ are the lateral strains of the CEPs, then the coordinates of the first and second CEPs are $\left(e_{1},\;\;\alpha \sigma_{\max}\right)$ and $\left(e_{2},\;\;\beta \sigma_{\max}\right)$, respectively. The rock-bearing capacity index (RockBCI) is defined as follows:

(2) $$BCI^{1}=\lambda \frac{e_{1}}{\alpha },\;\;BCI^{2}=\lambda \frac{e_{2}}{\beta }\;$$

where λ is the index amplification coefficient.

The RockBCI represents the hysteresis of CEP^1^ and CEP^2^. The hysteresis of CEP^1^ can describe the bearing capacity of rock samples at the peak stage, while the hysteresis of CEP^2^ can identify the bearing capacity of rock samples when the core-bearing capacity collapses.

**Figure 8:**
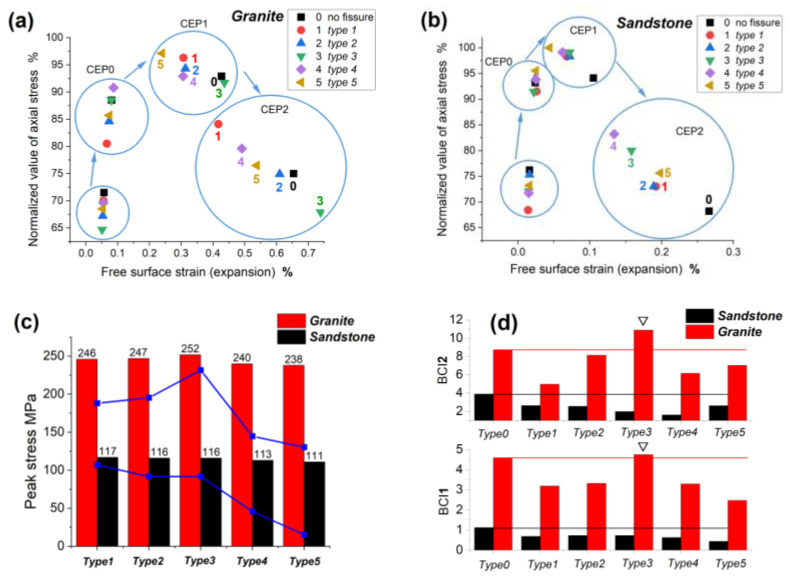
Characteristic points and indicators.

**Figure 9:**
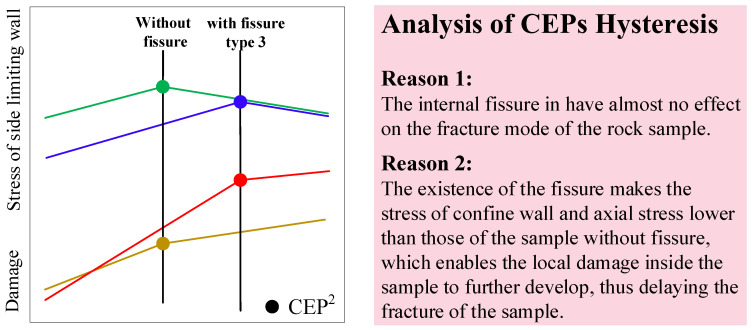
Analysis of the CEP hysteresis.

The CEPs and RockBCI provided a technical foundation for the quantitative analysis of the failure impact of fissures on the rock samples. Table 3 shows the coordinates of all the characteristic points of the rock samples in the 12 PSC tests. The abscissa is the lateral strain of free direction, and the ordinate is the normalized value of the axial stress. Additionally, the RockBCI of CEP^1^ and CEP^2^ is listed in Table 3, and its amplification coefficient λ was 1000. Figure 8a,b depicts the distribution of the characteristic points and the comparison of the axial peak stress and RockBCI of all rock samples.

a. **Peak stress comparison:**Figure 8c shows that type 5 and 4 fissures most significantly weakened the axial peak stress. The type 5 horizontal fissure caused an intense stress concentration because the fissure surface was perpendicular to the axial loading direction. Although the “beam” effect of the type 4 fissure was small, it caused the fracture surface of the sample to occur along the fissure surface, thus reducing the bearing capacity of the rock sample.
b. **Distribution of characteristic points:** The aggregation of four characteristic points was more evident in the granite samples, indicating that the stage process of the damage and collapse of the granite samples was more prominent. The short interval between the CEP^0^ cluster and the CEP^1^ cluster of sandstone indicated that the process of the bearing platform of sandstone was short. The sequence of each characteristic point varied between rock samples with various fissures. For rock samples with type 3 and type 1 vertical fissures, their DSP was the most forward and their CEP^2^ was the most backward. For rock samples with type 5 and type 10 horizontal fissures, their CEP^1^ was the most forward but their CEP^2^ was relatively lagging.
c. **Quantitative index analysis:** The sequence of the RockBCI^1^ was 3 > 2 > 4 > 1 > 5 (fissure type) for the granite samples and 3 > 2 > 1 > 4 > 5 for the sandstone. The sequence of the RockBCI^2^ was 3 > 2 > 5 > 4 > 1 for the granite samples and 1 > 5 > 2 > 3 > 4 for the sandstone. The bearing capacity of the granite samples at the peak and post-peak stages was identical. Three types of fissures most weakened the bearing capacity of the specimen: horizontal fissures, fissures whose angles coincided with the fracture surface, and fissures whose surface was perpendicular to the confine wall direction. In the peak stage, the bearing capacity of the sample with horizontal fissures was the worst, and in the fracture stage, the bearing capacity of the sample with 45° and 90° fissures was the lowest. The sequence of the RockBCI of CEP^1^ in the sandstone samples with fissures was identical to that of granite, whereas the sequence of the RockBCI of CEP^2^ in the sandstone samples was different from that of granite. CEP^2^ hysteresis was observed in the granite sample with the type 3 fissure.
d. **Analysis of CEP hysteresis:** The granite sample with a type 3 fissure was unique because its RockBCI of CEP^2^ was greater than that of the rock sample without any fissures, as shown in Figure 8d for the column that exceeds the red line. Figure 9 shows a simplified diagram of the stress of the confine wall and the damage trend under the situation, indicating that there are two reasons for the CEP hysteresis. First, the type 3 fissure had almost no effect on the fracture mode of the rock sample; second, the presence of the fissure reduced the stress of the confine wall and axial stress relative to a rock sample without any fissures, allowing for local damage within the sample to further develop and delay the fracture.

Accordingly, the DSP and CEPs showed the characteristics of aggregation distribution and sequence difference. The RockBCI was found to have a unified law that the horizontal fissures, fissures whose angle coincided with the fracture surface, and fissures whose surface was perpendicular to the lateral confine direction most weakened the bearing capacity of the specimen. It was discovered that a fissure type could delay a sample’s fracture as it did not affect the fracture mode but weakened the stress effect of the sample after the peak, allowing for the further development of local damage before a fracture occurred.

### 3.3. Crack Evolution

**Figure 10:**
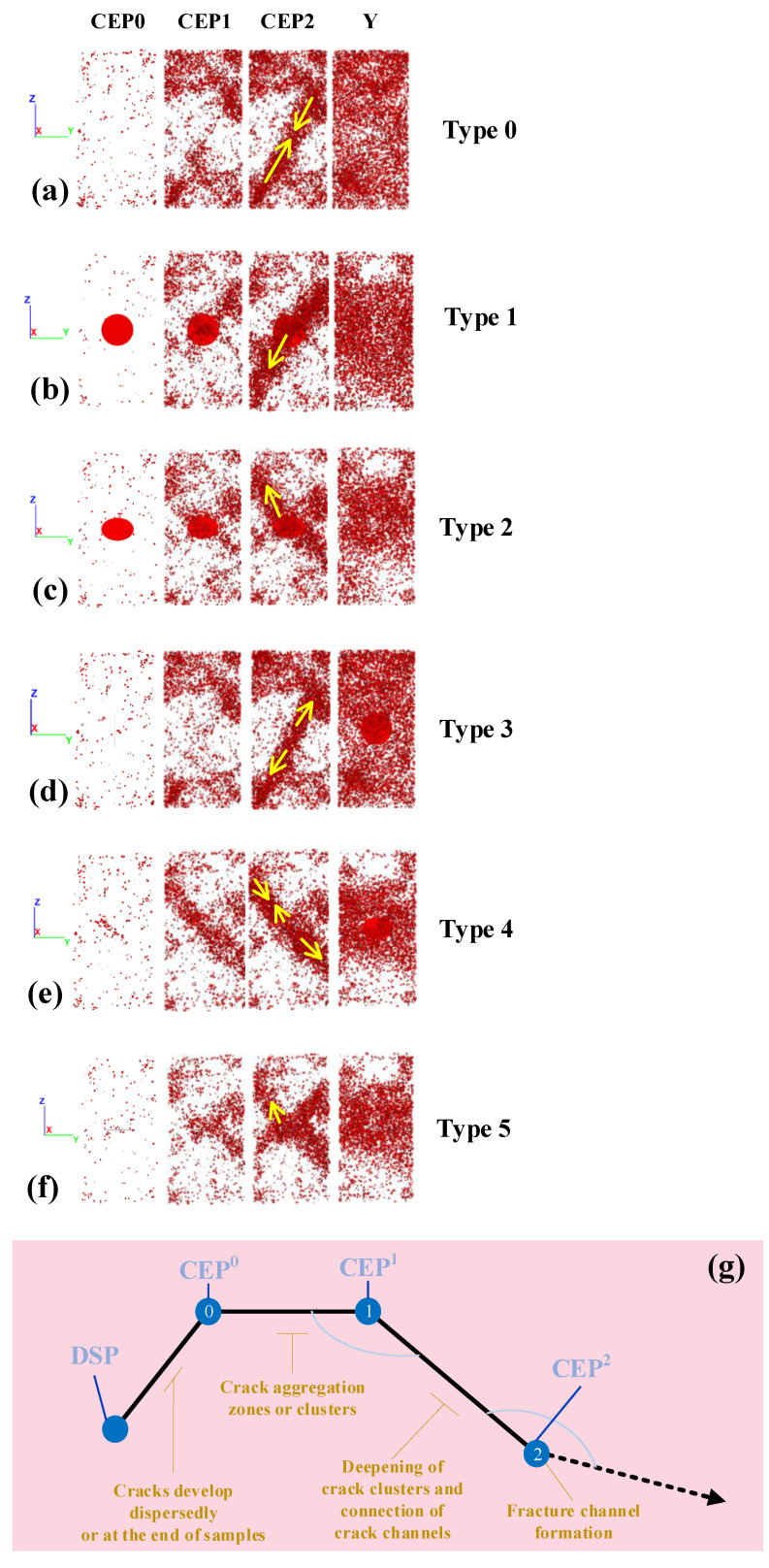
Crack development and evolution process.

With the advantage of the DEM, the development and evolution (D&E) of microcracks in the rock samples during PSC could be observed in real time. Figure 10a–f depicts the D&E state of microcracks (view of confine wall direction) at the time of the DSP and CEPs of the granite samples. Figure 8 indicates the following.

Generally, the D&E of cracks in the sample exhibited the unified localization law. With the compression loading from CEP^0^ to CEP^1^–CEP^2^, cracks grew from sporadic to local aggregation and then connected with other local aggregation cracks until the fracture was formed. Before CEP^0^, cracks uniformly and dispersedly developed or concentrated at the end of the sample. The CEP^0^–CEP^1^ of the load-bearing platform was the critical period for the local aggregation and development of cracks, which gradually formed crack aggregation zones or clusters, causing local damage but not fracture to the sample. The post-peak CEP^1^–CEP^2^ stage was the deepening period of local crack clusters and the connection period of the fracture channel. Finally, the specimen lost its core-bearing capacity at CEP^2^ immediately after forming the fracture channel, as revealed in Figure 10g.

The fissures in the rock samples significantly affected the D&E of the cracks. While the crack development was globally dispersed at CEP^0^, the inclined fissure of type 4 and the horizontal fissure of type 5 were the first to aggregate cracks around the fissure, indicating that these two types of fissures were most sensitive to pre-peak loading. At the stage of bearing platform CEP^1^–CEP^2^, the cracks of the sample without any fissures grew in a large number at the upper and lower part of samples before forming a shear fracture surface in the middle, which was the D&E path of ‘end→middle←end’. The cracks of the sample with fissures also developed from the upper and lower part at the beginning, but the internal fissure also became a starting position of crack development, forming the D&E path of ‘end ← fissure → end’.

Now, the time ratio index of CEPs (TRI) is further proposed. Let the TRI = Time of CEP^0^–CEP^1^/Time of CEP^1^–CEP^2^, i.e.,

(3) $$TRI=\frac{T_{0-1}}{T_{1-2}}$$

The TRI can describe the time ratio between the peak bearing phase, post-peak damage, and the collapse phase of the rock samples under PSC. When the TRI was greater, the specimen had a larger bearing capacity in the early damage stage but its destruction was more abrupt in the later fracture stage. In contrast, the smaller the TRI, the larger the specimen’s reserve time and signs before the final fracture.

**Figure 11:**
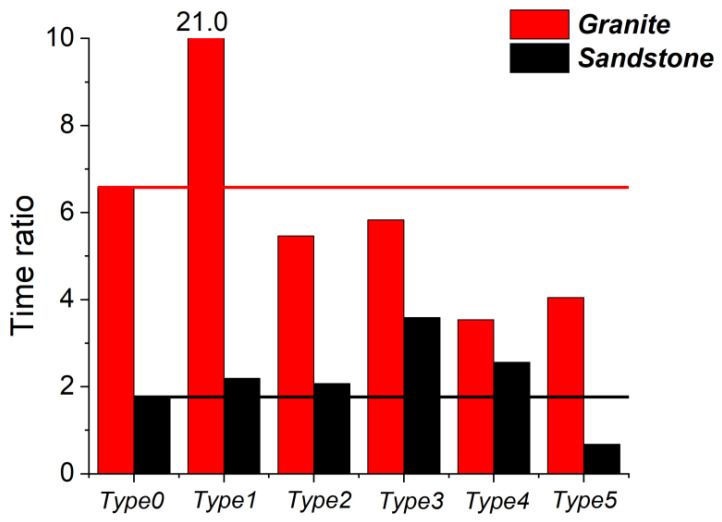
The time ratio index of CEPs.

Figure 11 illustrates the TRI of all the granite samples. It indicates that for granite samples, the TRI was the most prominent with the fissure types 1, 3, and 2—primarily type 1, whose TRI was three times that of the samples without fissures, indicating that the vertical fissure caused the samples to break faster and more suddenly under axial loading. For the sandstone samples, the TRI of fissure types 3 and 4 was higher than that of the samples without any fissures, demonstrating that the type of internal fissures generally made the bearing capacity of the sandstone samples more rapidly collapse.

Accordingly, the D&E of cracks followed the unified law of localization. Initially, cracks sporadically developed to gather locally and gradually formed crack clusters. The crack clusters then caused local damage to the rock, but the sample did not lose its core-bearing capacity. The crack clusters continued to deepen and gradually connect until the macro fracture formed. The existence of fissures not only changed the path of crack deepening connection, which was from the path of ‘end→middle’ to the ‘end←fissure→end’, but also modified the time process of the damage and fracture of the rock samples, causing the samples to more suddenly break after the peak or shortening the loading platform during the peak.

## 4. Conclusions

(1) Rock samples containing a particular fissure under PSC generally showed a weak face shear fracture mode, and the fracture mode was affected by the lithology, fissure angle, and fissure direction. Lithology was found to mainly affect the fracture angle. The fissure angle affected whether a rock sample was fractured along the fissure face. The fissure direction and its stress state determined whether stress concentration occurred around the fissure, which significantly impacted the bearing capacity of the rock sample.
(2) The CEPs of rock samples subjected to PSC were determined by analyzing elements of axial stress, confine wall stress, crack D&E trends, and the expansion of free surface (lateral strain). The damage start point, initial CEP, and first/second CEPs were the four CEPs. The two post-peak CEPs revealed the phase change of the damage and collapse of rocks under PSC. The RockBCI was proposed to represent the hysteresis of post-peak CEPs.
(3) The CEPs and RockBCI can provide a technical foundation for quantitative analysis of the failure effect of fissures on rock samples. In this quantitative analysis, all test samples’ peak stress and RockBCI values had a uniform law. The bearing capacity of the rock samples with horizontal fissures, fissures whose angles coincided with the fracture surface, and fissures whose surface was perpendicular to the lateral confine direction were the worst. Their BCI^2^ values were found to be 80.6%, 70.8%, and 56.9% of the rock samples without any fissures, respectively. There was a type of fissure that delayed the fracture. One reason was that the fissure did not affect the fracture mode; the second reason was that the fissure reduced the stress effect in the loading and confine direction.
(4) CEPs were used to divide the stages of the D&E of cracks, and the TRI was proposed to describe the time ratio between the CEP^0^–CEP^1^ and CEP^1^–CEP^2^ for rock samples under PSC. Cracks were found to develop from sporadic to local aggregation, gradually forming crack clusters. The crack clusters caused local damage to the rocks, but the specimens did not lose their bearing capacity until the local crack clusters continued to deepen and connect; then, macrocracks formed. The presence of internal fissures not only changed the path of crack deepening and connection but also modified the time course of rock damage and collapse.
(5) A limitation of this study is that there were no further physical experiments. As mentioned above, PSC tests of rock materials are difficult to implement. The key to a successful PSC test is to define the accuracy control conditions of the confining device. In fact, we allowed our confining device to have a deformation, but the plane strain state of the rock samples had to be guaranteed. In a follow-up study, we will propose accuracy control conditions of a confining device for rock specimens and reproduce the test discussed in this paper through physical experiments.

## Figures and Tables

**Table 1:** Parameter calibration law of the PFC model.

Lab Test Parameters (III)	PFC Model Parameters (5)				
	First Parameter: Effective Modulus	Second Parameter: Normal-to-Shear Stiffness Ratio	Third Parameter: Tension Strength of PBCM	Fourth Parameter: Cohesion of PBCM	Fifth Parameter: Friction Angle of PBCM
I/III Compression strength	Negligible	Weak relation	Positive correlation	Positive correlation	Positive correlation
II/III Elasticity modulus	Positive correlation	Power function decreasing relation	Negligible	Negligible	Negligible
III/III Poisson ratio	Negligible	Power function increasing relation	Negligible	Negligible	Negligible

**Table 2:** Parameter calibration test results.

	Granite			Sandstone		
	Compression Strength/MPa	Elasticity Modulus/GPa	Poisson Ratio	Compression Strength/MPa	Elasticity Modulus/GPa	Poisson Ratio
Lab test results	214.5	48.0	0.33	113.5	60.2	0.23
PFC test results	211.0	48.0	0.32	107.0	61.6	0.24
Error %	1.6	0.0	3.0	5.7	2.3	4.3

**Table 3:** Coordinates of CEPs and their RockBCI.

Granite	DSP	CEP^0^	CEP^1^	BCI^1^	CEP^2^	BCI^2^
Type 0	0.057 (71.5)	0.081 (88.5)	0.427 (92.9)	4.60	0.654 (75.0)	8.72
Type 1	0.055 (70.0)	0.066 (80.5)	0.307 (96.3)	3.19	0.417 (84.1)	4.96
Type 2	0.053 (67.2)	0.073 (84.6)	0.313 (94.3)	3.32	0.61 (74.9)	8.14
Type 3	0.051 (64.7)	0.080 (88.5)	0.436 (91.7)	4.75	0.739 (67.9)	10.88
Type 4	0.055 (69.6)	0.087 (90.8)	0.306 (92.9)	3.29	0.491 (79.6)	6.17
Type 5	0.053 (68.5)	0.074 (85.7)	0.240 (97.1)	2.47	0.538 (76.5)	7.03
Sandstone	DSP	CEP^0^	CEP^1^	BCI^1^	CEP^2^	BCI^2^
Type 0	0.016 (76.2)	0.024 (93.2)	0.105 (94.1)	1.12	0.266 (68.2)	3.90
Type 1	0.014 (68.4)	0.026 (91.5)	0.068 (98.3)	0.69	0.193 (73.0)	2.64
Type 2	0.016 (75.3)	0.024 (94.0)	0.072 (98.3)	0.73	0.189 (73.7)	2.56
Type 3	0.015 (72.1)	0.022 (91.4)	0.072 (99.1)	0.73	0.158 (80.0)	1.98
Type 4	0.015 (71.7)	0.025 (93.8)	0.062 (99.1)	0.63	0.134 (83.2)	1.61
Type 5	0.016 (73.2)	0.025 (95.5)	0.044 (100.0)	0.44	0.198 (75.6)	2.62

## Data Availability

Details of the results of individual tests can be provided by sending a request to J.W. (jqwen1994@163.com).
